# Renal resistive index for early prediction of acute kidney injury in sub-Saharan Africa: a scoping review protocol

**DOI:** 10.1136/bmjopen-2024-096093

**Published:** 2025-12-04

**Authors:** Busisiwe Mrara, Olubunmi Margaret Ogbodu, Olanrewaju Oladimeji

**Affiliations:** 1 Department of Anaesthesiology and Critical Care, Faculty of Medicine and Health Sciences, Walter Sisulu University, Mthatha, Eastern Cape, South Africa; 2 Public Health, Sefako Makgatho Health Sciences University Faculty of Health Sciences, Pretoria, Gauteng, South Africa

**Keywords:** Acute renal failure, End stage renal failure, Chronic renal failure

## Abstract

**Abstract:**

**Introduction:**

Acute kidney injury (AKI) is a complex, devastating condition characterised by a sudden reduction in renal function, leading to increased mortality and healthcare costs globally. Outcomes of AKI are worsened by factors such as limited access to healthcare, delayed hospital presentation and underlying comorbidities, which severely affect patients in sub-Saharan Africa. The renal resistive index (RRI) has come into view as an encouraging non-invasive imaging approach for the early prediction of AKI. However, the use of the RRI for AKI prediction in sub-Saharan Africa is poorly documented. This research aims to map and describe the evidence for using the RRI for the early detection of AKI in sub-Saharan Africa.

**Methodology:**

The Joanna Briggs Institute methodology for scoping reviews will be used for this study. It will include a comprehensive search of electronic databases, grey literature (including academic proceedings), as well as an extensive literature review of relevant journals. The Preferred Reporting Items for Systematic Reviews and Meta-Analyses for Scoping Reviews will also be used as a guide. Discrepancies will be handled by consensus or by consulting a third reviewer. This evidence synthesis will explore the usefulness and accuracy of the RRI for early prediction of AKI in sub-Saharan Africa, where the patients are generally younger and have different AKI risk predictors and cardiovascular profiles compared with patients in high-income countries. Evidence of implementation and associated challenges will also be explored. These challenges may include limited access to specialised ultrasound equipment and a lack of trained healthcare providers proficient in RRI measurement and interpretation. The findings will inform future studies and be useful for healthcare providers, policymakers and patient advocates seeking sustainable strategies for preventing AKI.

**Ethics and dissemination:**

Ethical approval is not required for this scoping review. The findings of this review will be published in a peer-reviewed journal and presented to decision-makers, health system administrators and healthcare providers at national and international academic conferences.

**STRENGTHS AND LIMITATIONS OF THIS STUDY**

- Our review will conform to the rigorous methodological guidelines for scoping review by the Joanna Briggs Institute.
- Application of a well-established methodological framework will ensure the production of a high-quality review of evidence.
- The inclusion of grey literature strengthens our review by reducing publication bias and enhancing the comprehensiveness of our findings.
- Omission of studies un-indexed in databases and consulted grey literature.
- The synthesis will be limited to articles published in English; hence, some evidence in languages other than English may be missed.

## Introduction

 Acute kidney injury (AKI) is a significant public health issue in sub-Saharan Africa and worldwide, with multiple recorded cases and poor clinical outcomes.^1^ The condition is characterised by a sudden reduction in renal function, leading to increased morbidity and mortality.^2^ The incidence of AKI in sub-Saharan Africa ranges from 10% to 30% in hospitalised patients, with mortality rates as high as 60%.^3^

Timely diagnosis and intervention are essential in mitigating the severe outcomes of AKI. However, this is not always possible in sub-Saharan Africa due to various challenges, including limited access to diagnostic tools and specialised healthcare services.^4^ Diagnostic methods, such as serum creatinine and urine output, currently in use, perform sub-optimally in detecting AKI in its early stages.^5^ Therefore, there is a pressing need for innovative biomarkers and diagnostic imaging tools to enhance early prediction and management of AKI.

In this context, the use of renal resistive index (RRI) has emerged as a promising non-invasive approach for the early prediction of AKI. The RRI is a non-invasive Doppler ultrasound-based measure of intrarenal vascular haemodynamics.^6^ The RRI is supported by good evidence as a tool for the early identification of AKI, as it can detect changes in renal haemodynamics before the clinical manifestation of kidney injury.^7^ In addition, the RRI is effectively used to differentiate reversible AKI from persistent AKI.^7^ By measuring the resistance to blood flow within the intrarenal arteries, RRI can provide valuable information about renal perfusion, potentially enabling clinicians to intervene by optimising intravenous fluids and haemodynamics before the development of overt AKI.

The RRI is derived from blood flow velocities measured in the interlobar arteries within the renal parenchyma, where the index is computed using the formula (peak systolic velocity − end diastolic velocity)/peak systolic velocity.^6^ From this formula, RRI is an indicator of the relationship between renal parenchymal blood flow velocity and the ratio of systolic to diastolic measurements. The RRI has been described as an indicator of renal vascular resistance with a correlation with renal vascular compliance and systemic haemodynamics, all of which have an impact on renal perfusion.^7 8^ In this regard, the RRI is influenced not only by renal vascular tone but also by systemic blood pressure, heart rate, vessel structure and endothelial function, which have been shown to impact vascular compliance. Vascular compliance is additionally affected by age and comorbidities such as diabetes and hypertension, which affect the endothelium and vessel walls.^9^ RRI values higher than 0.7 have been associated with an increased risk of AKI and renal dysfunction^10 11^; however, it is unclear if these values should be extrapolated across all ages and cardiovascular profiles. Patients presenting with AKI in sub-Saharan Africa are younger compared with high-income countries (HIC) patients with a lower burden of non-communicable disease; therefore, the performance of the RRI in sub-Saharan Africa could be different from HIC.

Preliminary literature searches reveal the application of RRI in various clinical settings, including the evaluation of hydronephrosis and renovascular hypertension in sub-Saharan Africa. However, the evidence on the usefulness of RRI in AKI prediction in sub-Saharan Africa is limited. A pilot study by Lintner *et al*, in which RRI was unable to predict AKI in paediatric patients with cerebral malaria, is evidence of the variable performance of the RRI in AKI prediction across age groups.^12^ A comprehensive synthesis of the available data is needed to assess its potential for early AKI prediction in this region.

Health systems in sub-Saharan Africa also have challenges, such as limited access to specialised ultrasound equipment.^13 14^ The lack of trained healthcare providers proficient in RRI measurement and interpretation is also a possibility in limited-resource settings.^15^

The overall goal of this scoping review is to systematically and comprehensively map what is known, understand the studies and the existing evidence on the use of RRI for the early prediction or detection of AKI in populations within sub-Saharan Africa. This scoping review intends to synthesise the existing literature review on RRI for early prediction of AKI in sub-Saharan Africa, with the following research objectives:

- To ascertain the state of evidence of RRI for early detection of AKI in sub-Saharan Africa.
- To explore the barriers to implementing RRI for AKI prediction in sub-Saharan Africa, focusing on health system deficiencies, including equipment and human resources.

The primary outcome of this scoping review is to map and characterise existing evidence on the use of RRI for the early prediction or diagnosis of AKI in populations within sub-Saharan Africa. The secondary outcomes of this review will include a description of the measurement characteristics of RRI, including patient demographics and comorbidities, the association between RRI and AKI severity, and clinical outcomes such as mortality, renal replacement therapy, and gaps and limitations in current evidence, including geographical or population gaps within sub-Saharan Africa, health system or resource limitations influencing RRI use.

Overall, this scoping review aims to provide a comprehensive overview of the current state of knowledge, identify knowledge gaps and inform future research priorities in this critical area.

## Methods

This scoping review will be conducted in accordance with the Joanna Briggs Institute methodology for scoping reviews.^16^ The Preferred Reporting Items for Systematic Reviews and the Meta-Analyses for Scoping Reviews (PRISMA-ScR) will serve as its guidelines.^17^ The review will use a systematic method to identify, select and synthesise relevant studies on the use of RRI for the early detection of AKI in sub-Saharan Africa.

### Search strategy

A comprehensive search strategy will be developed, incorporating critical terms related to AKI, RRI and sub-Saharan Africa. Electronic databases, including PubMed, Scopus, ScienceDirect and Google Scholar, will be consulted for the needed studies. The comprehensive database search strategy for this review is provided as online supplemental table 1), uploaded with this manuscript. Additionally, grey literature from academic proceedings and relevant organisational websites will be sourced for unpublished and ongoing research. The search terms will include the keywords or Medical Subject Headings: renal resistive index, acute kidney injury, early prediction, renal Doppler ultrasonography, kidney function and sub-Saharan Africa. The search strategy will be piloted to assess the appropriateness of keywords and databases, as well as the use of Boolean operators. A manual search will also be conducted for publications from sub-Saharan African countries.

### Study selection

Two independent reviewers will screen the headings and abstracts of the retrieved literature using the PICO (Population Intervention Comparator Outcomes) framework to identify studies that qualify for the following inclusion criteria. The PICO framework is shown in table 1 below.

**Table 1:** PICO framework for identification of studies

Population	Patients in sub-Saharan Africa
Intervention	Use of RRI for the early detection of AKI
Comparator	Any comparator or no comparator
Outcome	Diagnostic accuracy of RRI for AKI prediction Factors influencing RRI performance Barriers and facilitators to RRI implementation

AKI, acute kidney injury; PICO, Population Intervention Comparator Outcomes; RRI, renal resistive index.

A standardised data extraction form will be used to retrieve essential data from the qualified literature, including study characteristics, patient demographics, RRI measurement methods, AKI definitions and diagnostic test performance. The extracted data will be narratively synthesised to address the research questions, focusing on identifying patterns, trends and gaps in the existing evidence.

### Eligible study selection

The following criteria in table 2 will be used to select relevant studies.

**Table 2:** Inclusion and exclusion criteria

Inclusion criteria	Exclusion criteria
Research written in English	Research not written in English
Studies that examine the use of RRI for the prediction of AKI in sub-Saharan Africa	Studies that do not examine the use of RRI for the prediction of AKI
Studies published between January 2010 and December 2024	Studies published before 2010 or after 2024
Studies conducted in sub-Saharan Africa	Studies conducted outside of sub-Saharan Africa

AKI, acute kidney injury; RRI, renal resistive index.

### Data extraction

**Figure 1:**
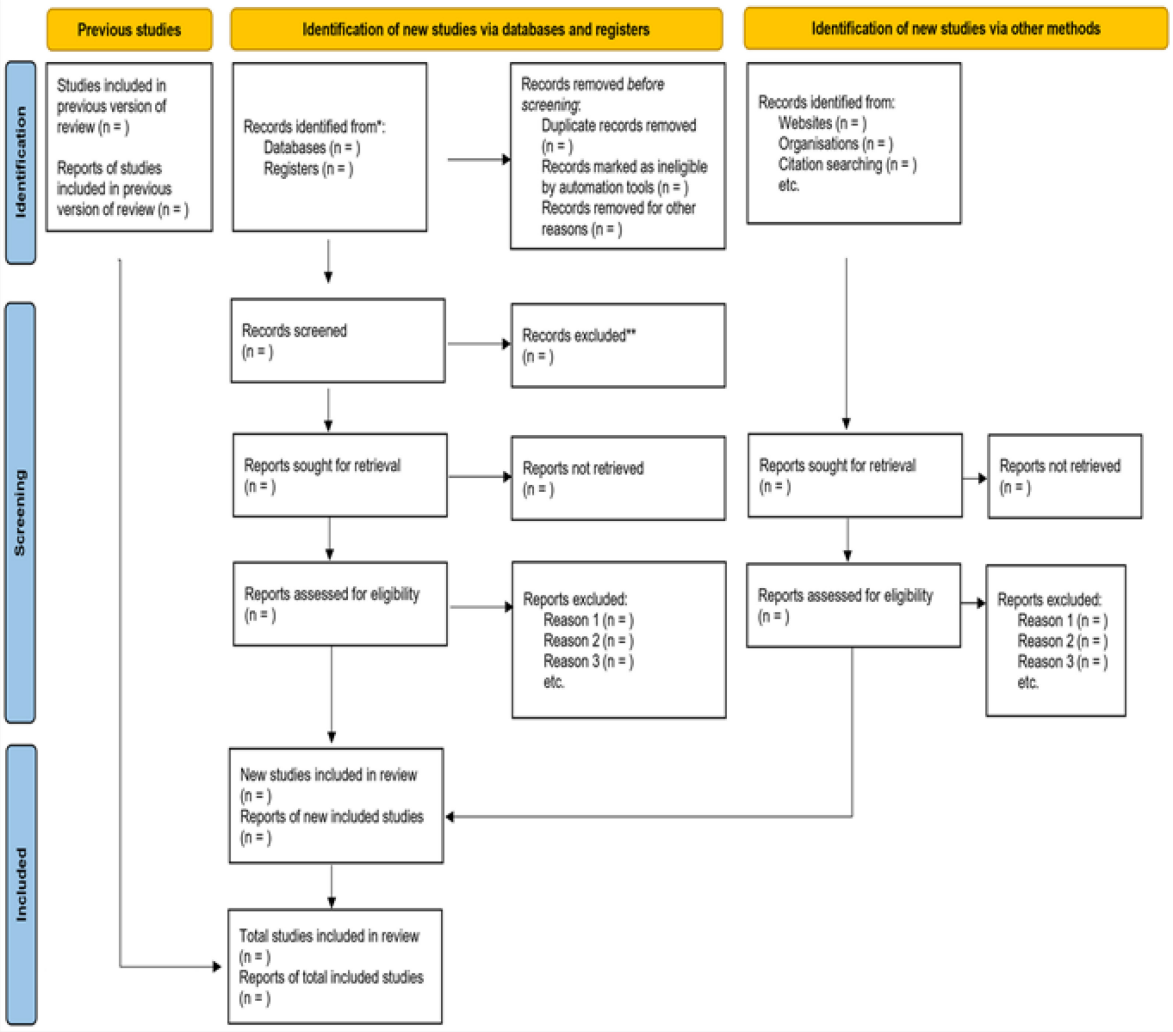
Preferred Reporting Items for Systematic Reviews and meta-analysis for Scoping Reviews flowchart for eligible study selection.^17^

The review group will create and test a standardised data-extracting form. The following information will be drawn out from the included research: study characteristics, population characteristics, RRI measurement methods, AKI definition and diagnosis, diagnostic accuracy measures, factors influencing RRI performance, and barriers and facilitators to the implementation of RRI for AKI prediction. The outline of the data extraction decisions will be duly followed using the PRISMA-ScR flow chart, as shown in figure 1.^11 17^

### Charting the data

The extracted information will be charted using a standardised figure to facilitate data synthesis and analysis. The detailed information will be organised in a table format, as shown in box 1, to provide an overview of the included studies and facilitate the synthesis of the evidence. The data will be charted in accordance with the research questions and a description of key findings, trends and gaps in existing literature.

Box 1 **Data charting**

Author and year of publication

Journal

Aim of study

Study population

Study setting (rural and urban)

Study location (Africa)

Study design

Main findings

Other significant findings

### Collating, summarising and reporting the results

The charted data will be collated, summarised and reported in a narrative format, highlighting the key findings, methodological limitations and clinical implications. The extracted data will be narratively synthesised to address the research questions. The findings will be presented in a clear and structured manner, with tables, figures and narrative descriptions, as appropriate.

### Quality assessment

The quality of the inclusion research will be examined using the Newcastle-Ottawa Scale for observational studies and the Quality Assessment of Diagnostic Accuracy Studies tool for diagnostic accuracy studies.^18^ The negative outcome of bias will be investigated using the Cochrane Risk of Bias Tool.^19^ By using a systematic and comprehensive approach, this scoping review aims to establish a thorough synthesis of the present evidence on RRI for early prediction of AKI in sub-Saharan Africa, highlighting the gaps and areas for future research. Eligible studies in English in the period 2010–2024 will be applied to the search. The rationale for the literature search period, 2010–2024, is that the RRI gained significant scientific research traction in this period, particularly as a promising, non-invasive tool for predicting AKI, especially in critically ill patients and post surgery. During this period, research publications reported that RRI performed better in early detection than traditional markers of AKI, such as creatinine. Even though global organisations have not yet established formal guidelines or made official declarations regarding RRI for AKI diagnosis, there have been numerous research studies and clinical observations published in peer-reviewed journals that support the use of RRI as an early predictor of AKI, particularly after cardiac surgery, in the intensive care unit and in other patient groups.^20 21^

Additionally, the bibliography details of inclusion research and relevant reviewed studies will be manually sourced to identify any additional research that may have been overlooked during electronic sourcing.

Full-text studies will then be retrieved and independently examined by the two investigators after selection. Any discord between the investigators will be addressed through open communication or, if necessary, by involving an additional investigator to provide further insight, facilitate discussion and reach a consensus.

## Discussion

This scoping review on the use of the RRI for the early detection of AKI in sub-Saharan Africa aims to provide insights into the current state of evidence for this research and describes the potential for optimising patient care in AKI prevention in the region.

A critical review of the literature will be conducted to understand the factors that may influence the performance of RRI in predicting AKI, the nuances of RRI interpretation, and its applicability in diverse clinical settings within sub-Saharan Africa, with the aim of enhancing the predictive value of RRI in AKI. A preliminary literature search identified several factors that may generally influence the performance of RRI in predicting AKI. Patient-related factors, such as age, comorbidities and the aetiology of AKI, were found to affect the diagnostic accuracy of the RRI.^20 21^

Additionally, the literature search will highlight the importance of standardising RRI measurement techniques, including a description of variations in ultrasound equipment, probe positioning and operator expertise, as well as how these factors can affect the reliability and reproducibility of RRI values. These findings will underscore the need for comprehensive training and quality assurance programmes in place to ensure the consistent and accurate use of RRI in healthcare settings. Furthermore, developing and validating context-specific RRI reference ranges and decision thresholds could significantly enhance the use of this diagnostic tool of RRI in the sub-Saharan region.

The existing evidence of the use of renal Doppler RRI for the early diagnosis of AKI in sub-Saharan Africa is a crucial topic in the field of nephrology, given that AKI is associated with excess morbidity and mortality rates, and inadequate resources for renal replacement therapy and transplantation. The growing availability of portable and affordable ultrasound devices in sub-Saharan Africa, combined with the increasing recognition of the burden of AKI in the region, presents opportunities for evaluating the role of RRI in improving early detection and reducing poor outcomes related to AKI, a gap that this study aims to address.

This scoping review will also help identify potential barriers and facilitators for the widespread implementation of RRI in AKI prediction in sub-Saharan Africa, including, but not limited to, access to specialised ultrasound equipment, training, healthcare provider expertise and clinical guidelines or protocols on RRI use. It will also inform the development of context-appropriate guidelines and protocols that could significantly enhance the implementation of RRI for AKI prediction in sub-Saharan Africa.

### Ethics and dissemination

Ethical approval is not required for this scoping review. The findings of this review will be published in a peer-reviewed journal and presented to decision-makers, health system administrators and healthcare providers at national and international academic conferences.

## Supplementary material

10.1136/bmjopen-2024-096093 online supplemental file 1
