# Supplementary material for: Renal resistive index for early prediction of acute kidney injury in sub-Saharan Africa: a scoping review protocol
Source: BMJ Open. 2025 Dec 4;15(12):e096093. doi: 10.1136/bmjopen-2024-096093 (PMC12684207; doi:10.1136/bmjopen-2024-096093)
Supplement: online supplemental file 1 [file bmjopen-15-12-s001.docx]

**Electronic Database Search strategy for Scoping Review Protocol manuscript on Acute Kidney Injury in the BMJ Open journal**

| **Database** | **Search String** | **Search results** |
| --- | --- | --- |
| **PubMed**  **Filters applied: Abstract, Free full text, Full text, from 2010/1/1 - 2024/12/31** | ("Renal Insufficiency"[MeSH Terms] AND "Sub-Saharan Africa"[Text Word]) AND ((ffrft[Filter]) AND (fha[Filter]) AND (fft[Filter]) AND (2010/1/1:2024/12/31[pdat]))  ("Renal Insufficiency"[MeSH Terms] AND "Sub-Saharan Africa"[Text Word]) AND ((ffrft[Filter]) AND (fha[Filter]) AND (fft[Filter]) AND (2010/1/1:2024/10/20[pdat])) | 131  129 |
| **Scopus**  **Filters applied:**  **Date range: 2010 - 2024**  **Filter by subject areas - Medicine, Multidisciplinary**  **Immunology and Microbiology, Biochemistry, Genetics and Molecular Biology, Pharmacology, Toxicology and Pharmaceutics, Nursing, Agricultural and Biological Sciences, Health Professions, Neuroscience, Materials Science,**  **Environmental Science, Computer Science,**  **Social Sciences, Physics and Astronomy,**  **Engineering, Earth and Planetary Sciences,**  **Chemistry, Chemical Engineering, Arts and Humanities** | TITLE-ABS-KEY ("Acute Kidney Injury") AND  ("Sub-Saharan Africa") | 410 |
| **ScienceDirect**  **Filters applied: Years, Custom range: 2010-2024**  **Subject areas: Medicine and Dentistry, Agricultural and Biological Sciences, Veterinary Science and Medicine** | Acute Kidney Injury, Renal Resistive Index, Renal Doppler ultrasonography, Sub-Saharan Africa.  Acute Kidney Injury, Early prediction, Renal Resistive Index, Renal Doppler ultrasonography, Sub-Saharan Africa. | 16  10 |
| **Google Scholar**  **Filters applied:**  **Custom range: 2010 – 2024** | “The use of renal resistive index in Acute Kidney Injury in Sub-Saharan Africa”  “The use of renal resistive index in Acute Kidney Injury in Sub-Saharan Africa” | 12,300  (any type of article)  2,250 (review articles only) |
