# Supplementary material for: Renal resistive index for early prediction of acute kidney injury in sub-Saharan Africa: a scoping review protocol
Source: BMJ Open. 2025 Dec 4;15(12):e096093. doi: 10.1136/bmjopen-2024-096093 (PMC12684207; doi:10.1136/bmjopen-2024-096093)
Supplement: Supplementary file 2 [file bmjopen-2024-096093.reviewer_comments.pdf]

PEER REVIEW HISTORY

BMJ Open publishes all reviews undertaken for accepted manuscripts. Reviewers are asked to complete a checklist review form and are provided with free text boxes to elaborate on their assessment. These free text comments are reproduced below.

ARTICLE DETAILS

Title (Provisional)

Renal Resistive Index for Early Prediction of Acute Kidney Injury in Sub-Saharan Africa: A Scoping Review Protocol

Authors

Mrara, Busisiwe; OGBODU, OLUBUNMI Margaret; Oladimeji, Olanrewaju

---

VERSION 1 - REVIEW

**Reviewer**	**1**
**Name**	**Carollo, Caterina**
**Affiliation**	**University of Palermo, PROMISE**
**Date**	**26-Mar-2025**
**COI**	**None**

Dear Authors,

Thanks for your manuscript which covers an important field of research. I appreciated the topic, the methods and discussion. Introduction is worthy of enlargement with demographigal,

---

**Reviewer**	**2**
**Name**	**Romano, Giulio**
**Affiliation**	**University of Udine**
**Date**	**23-Jul-2025**
**COI**	**None**

The authors' proposed goal, using the Renal Resistive Index for the early detection of acute kidney injury, represents a very useful objective. It is a non-invasive and costly test, excellent for use, especially in clinical settings where it may be difficult to evaluate the trend of equally early biohumoral biomarkers, but difficult to measure, to identify (and therefore prevent) acute kidney injury early.

**Reviewer**	**3**
**Name**	**Fiorentino, Marco**
**Affiliation**	**University of Bari**
**Date**	**05-Oct-2025**
**COI**	**None**

The present manuscript aims to describe and report the protocol of a scoping review focusing on the impact of RRI for AKI prediction in a specific subset of patients (Sub-saharian africa).

Although it is well written and no methodological issues have been detected, this reviewer does not think that the publication of this scoping review protocol is suitable for publication in BMJ open, since a clear definition of the goals of this analysis are not sistematically reported.

VERSION 1 - AUTHOR RESPONSE

Reviewer: 1

Dr. Caterina Carollo, University of Palermo

Comments to the Author:

Dear Authors,

Thanks for your manuscript which covers an important field of

research. I appreciated the topic, the methods and discussion.

Introduction is worthy of enlargement with demographigal,

Authors' Response: Thank you for reading our manuscript. The reviewer's comment is incomplete, as it is cut off, so we are unable to respond to it.

Reviewer: 2

Dr. Giulio Romano, University of Udine

Comments to the Author:

The authors' proposed goal, using the Renal Resistive Index for the early detection of acute kidney injury, represents a very useful objective. It is a non-invasive and costly test, excellent for use, especially in clinical settings where it may be difficult to evaluate the trend of equally early bio-humoral biomarkers, which are difficult to measure and identify (and therefore prevent) acute kidney injury early.

Authors' Response: Thank you for reading and reviewing our manuscript. We appreciate your feedback on the study objective.

Reviewer: 3

Dr. Marco Fiorentino, University of Bari

Comments to the Author:

The present manuscript aims to describe and report the protocol of a scoping review focusing on the impact of RRI for AKI prediction in a Specific subset of patients (Sub-Saharan Africa). Although it is well written and no methodological issues have been detected, this reviewer does not think that the publication of this scoping review protocol is suitable for publication in BMJ Open, since a clear definition of the goals of this analysis is not systematically reported.

Authors' Response: Thank you for your feedback. We have provided a clear definition of the goals along with the primary and secondary outcomes of this review in the revised manuscript.
